# Supplementary material for: Analysis of PPARγ Signaling Activity in Psoriasis
Source: Int J Mol Sci. 2021 Aug 10;22(16):8603. doi: 10.3390/ijms22168603 (PMC8395241; doi:10.3390/ijms22168603)
Supplement: Supplementary file 1 [file ijms-22-08603-s001.zip › Supplemental materials_Analysis of PPARg signaling activity in psoriasis/Pathway models/Models images and html files/PPARG inhibits phenotypes assotiated with psoriasis/240684.html]

psoriatic arthritis


# Disease psoriatic arthritis

|  |  |
| --- | --- |
| URN | urn:agi-meshdis:Arthritis%2c%20Psoriatic |
| Total Entities | 1 |
| Connectivity | 1078 |
| Name | psoriatic arthritis |

---

|  |  |
| --- | --- |
| ChildConcepts | juvenile psoriatic arthritis |

---

|  |  |
| --- | --- |
| Pathway | Proteins Involved in Psoriatic Arthritis |
|  | Genes with Mutations Associated with Psoriatic Arthritis |
|  | Proteins with Altered Expression in Psoriatic Arthritis |
|  | PPARG inhibits phenotypes assotiated with psoriasis |

---

|  |  |
| --- | --- |
| MedScan ID | 9004432 |

---

|  |  |
| --- | --- |
| Alias | Psoriatic Arthropathies |
|  | psoriatic rheumatoid arthritis |
|  | Arthritis, Psoriatic |
|  | Arthropathic Psoriasis |
|  | Psoriatic Arthritis |
|  | arthritis psoriatica |
|  | Psoriatic Arthropathy |
|  | alibert bazin disease |
|  | psoriatic polyarthritis |
|  | psoriatic rheumatism |
|  | Psoriasis, Arthritic |
|  | Arthropathy Psoriatic |
|  | Arthritic Psoriasis |
|  | psoriasis pustulosa arthropathica |
|  | Psoriasis with Arthropathy |
|  | Psoriasis, Arthropathic |
|  | Psoriasis Arthropathica |

---

|  |  |
| --- | --- |
| MeSH ID | D015535 |
|  | M0023901 |

---

|  |  |
| --- | --- |
| MeSH Heading | Arthritis, Psoriatic |

---
